# International Knee Documentation Committee Subjective Knee Form Latent Growth Model Analysis: Assessing Recovery Trajectories

**DOI:** 10.3390/healthcare12101021

**Published:** 2024-05-15

**Authors:** Katrina Dowell, Alexandra Dluzniewski, Madeline P. Casanova, Caleb M. Allred, Adam C. Cady, Russell T. Baker

**Affiliations:** 1 WWAMI Medical Education Program, University of Washington School of Medicine, Seattle, WA 98195, USA; krdowell@uw.edu; 2 WWAMI Medical Education Program, University of Idaho, Moscow, ID 83844, USA; adluz@uidaho.edu (A.D.); mcasanova@uidaho.edu (M.P.C.); cmallred@uw.edu (C.M.A.); 3 Idaho Office of Underserved and Rural Medical Research, University of Idaho, Moscow, ID 83844, USA; 4 Kaiser Permanente, Woodland Hills, CA 91367, USA; adam.c.cady@kp.org

**Keywords:** knee pathology, latent growth modeling, patient-reported outcome measure

## Abstract

Patient-Reported Outcome Measures (PROMs), such as the six-item International Knee Documentation Committee Subjective Knee Form (IKDC-6), play a crucial role in assessing health conditions and guiding clinical decisions. Latent Growth Modeling (LGM) can be employed to understand recovery trajectories in patients post-operatively. Therefore, the purpose of this study was to assess LGM properties of the IKDC-6 in patients with knee pathologies that require surgical intervention and to assess differences between subgroups (i.e., sex and age). A cross-sectional study was conducted using the Surgical Outcome System (SOS) database with patients who had undergone knee arthroscopy. Our results found that preoperative scores did not influence the rate of change overtime. Perceived knee health improved over time, with varying rates among individuals. The adolescent age subgroup and male subgroup exhibited faster recovery rates compared to the older age subgroup and female subgroup. While initial hypotheses suggested IKDC-6 could serve as a prognostic tool, results did not support this. However, results indicated favorable outcomes irrespective of preoperative perceived knee impairment levels. This study provides valuable insights into recovery dynamics following knee surgery, emphasizing the need for personalized rehabilitation strategies tailored to individual patient characteristics.

## 1. Introduction

Approximately 14 million Americans suffer from knee pathologies (e.g., osteoarthritis), which have profound health consequences (e.g., depression, cardiovascular disease, reduced quality of life) due to physical limitations. Patient Reported Outcome Measures (PROMs) have grown in importance in the last several decades [1,2] and are used to measure patients’ perspectives on various biopsychosocial variables (e.g., knee health, depression) [3,4] and may provide critical information that may influence both empirical and clinical decision-making [5,6,7,8] over the course of medical treatment and recovery. Therefore, using PROMs to better understand how individuals recover from various knee pathologies is essential for evaluating patient-perceived healing and making clinical decisions postoperatively.

Psychometric validity is paramount when assessing PROMS, ensuring that the measure effectively captures what it intends to assess. Rigorous validation processes, often involving statistical analyses and clinical evaluations, establish the PROM’s credibility and trustworthiness, ultimately enhancing their utility in informing clinical decisions, evaluating interventions, and improving patient-centered care. Further, once validity and reliability have been established, PROMs can be used for hypotheses testing (e.g., subgroup analyses, change over time) [9]. One type of analysis that can be used to assess change over time is Latent Growth Modeling (LGM) [9]. LGM is an analytical approach that allows examination of individual and group differences in terms of rate of change as well as assessment of which variables influence the rate of development [10]. The clinical course of knee pathology is difficult to predict; however, LGM can be used to identify trajectories and changes in physical health over time [9,11].

The International Knee Documentation Committee Subjective Knee Form (IKDC-SKF), an internationally recognized PROM, is widely used by healthcare societies to assess knee pathologies in orthopedics and sports medicine [12,13,14,15,16] and has been translated for use in multiple languages [14,15,17,18,19,20,21]. The IKDC-SKF was proposed as a 19-item model, assessing symptoms (e.g., pain, stiffness), sport activity (e.g., stair ambulation, knee movement), and function (e.g., daily activities); however, researchers have recently proposed the use of condensed versions of the scale due to improved model fit and decreased patient response burden [22,23]. For example, three short-form versions of the IKDC-SKF (six-, eight-, and nine-items) have been identified using Exploratory Factor Analysis (EFA) and Confirmatory Factor Analysis (CFA) procedures on large (n > 1000) datasets [22]. All three short-form versions have evidence of multigroup and longitudinal invariance, supporting their use to assess group differences and change over time [22]; however, the six-item IKDC-SKF (i.e., IKDC-6) was recommended due to greater model precision [22]. Because longitudinal invariance was established for the six-item IKDC-SKF [22], LGM can be used to guide assessment of patient recovery.

Assessing the IKDC-6 to better understand recovery rate in patients with multiple knee pathologies by using LGM procedures has not been completed. This analysis would allow classification of individuals into distinct groups based on trajectory across time [24,25]; thus, clinicians could have better insights into the likely trajectory for a patient’s recovery to support personalized care in order to maximize individual functioning and minimize future disability. Therefore, this study’s purpose was to assess the rate of change in patients recovering from knee arthroscopy by conducting LGM analysis on the IKDC-6 to determine if the IKDC-6 can be used as a prognostic tool and to examine if sex (i.e., male, female) and age groups (i.e., adolescents, emerging adulthood, young adulthood, middle adulthood) recover at different rates. We hypothesized that the IKDC-6 could be used as a prognostic tool, that significant differences between male and female recovery rate would not be identified, and that a significant difference in recovery rate between age groups would be found.

## 2. Materials and Methods

### 2.1. Data Source

The Surgical Outcome System (SOS) is an international, deidentified patient-reported outcome database that adheres to the Health Insurance Portability and Accountability Act (HIPPA). The SOS can be used for retrospective analysis, with the collected data deriving from patients who provided informed consent for data use. Cedar-Sinai Office of Research Compliance and Quality Improvement granted IRB approval as part of a larger research project using SOS data. The dataset used included IKDC-SKF responses at four time points: (1) baseline, prior to receiving care (i.e., knee arthroscopy), (2) 3 months post-intervention, (3) 6 months post-intervention, and (4) 1 year post-intervention.

### 2.2. Instrumentation

The IKDC-6 [22] is an adapted version of the IKDC-SKF. Example items include “What is the highest level of activity that you can perform without significant knee pain?”, “What is the highest level of activity you can perform without significant swelling in your knee?”, “What is the highest level of activity you can participate in on a regular basis?”; individuals rate items using a 5-point Likert scale (4 = very strenuous activities like jumping or pivoting as in basketball or soccer; 3 = strenuous activities like heavy physical work, skiing, or tennis; 2 = moderate activities like moderate physical work, running, or jogging; 1 = light activities like walking, housework, or yard work; 0 = unable to perform any of the above activities due to knee pain). An additional three-part question—“How does your knee affect your ability to: go upstairs, squat, sit with your knee bent”—is rated on a 5-point Likert scale with 4 indicating “not difficult at all” and 0 indicating “unable to do”. The IKDC-6 is calculated as the (sum of the completed items)/(maximum possible sum of the completed items) multiplied by 100.

### 2.3. Data Analysis

All data and demographic information were extracted from the SOS database, imported into Excel, and uploaded to the Statistical Package for Social Sciences (IBM SPSS Statistics for Windows, Version 27.0. Armonk, NY, USA: IBM Corp) and Analysis of Moment Structure (AMOS, SPSS, Inc. Armonk, NY, USA: IBM Corp) version 27 for data analysis. Any individuals missing demographic variables remained in the dataset. Individuals missing more than 10% of the IKDC-6 (i.e., two of the six items) at any of the four time points were removed from the dataset. Individuals who had one missing item on the IKDC-6 had the missing value replaced using mean imputation [9]. After the missing data were treated, descriptive statistics were calculated to obtain means, standard deviations, and frequencies. Further, data normality was assessed using skewness (|2|) and kurtosis values (|7|) [26,27]. Univariate and multivariate outliers were examined with z-scores of |3.3| and Mahalanobis distance with chi-square values of *p* < 0.01 [9,28].

#### Latent Growth Modeling

An LGM model was conducted on the IKDC-6. Goodness-of-fit indices [27] for each model were evaluated using the following guidelines [9,27,29]: Comparative Fit Index (CFI ≥ 0.95), Tucker–Lewis Index (TLI ≥ 0.95), Standardized Root Mean Square Residual (SRMR ≤ 0.08), Root Mean Square Error of Approximation (RMSEA ≤ 0.06), and Bollen’s Incremental Fit Index (IFI ≥ 0.95. Additionally, while the likelihood ratio statistic (CMIN) was assessed, it is not as informative as the other fit indices due to it being overly sensitive to sample size [9,26]. The LGM was then assessed for linear and non-linear growth by setting the slope factor loadings to 0, 1, 2, 3 for linear growth and 0, 1, 3, 9 for quadratic growth. Along with the slope factor, the intercept factor was specified to have a factor loading of 1, meaning that the intercept value remained constant across time for each individual [27]. After a well-fitting model was established, the following parameters were evaluated: the means of the intercept and slope factors with their related variances and covariances [27]. The LGM parameters provide the following information: (a) intercept values represent the individual’s score on the outcome variable at the initial time point, (b) slope values represent the individual’s rate of change over time, (c) mean scores for the intercept and slope represent the average values for the sample, ergo, where the sample starts, and the average growth trajectory over time, and (d) variance for the intercept and slope values represent how much individuals deviate from the sample at the initial time point and growth trajectories over time [9,27]. Lastly, covariance values represent deviation in the initial status and rate of change in the sample, explaining how the initial score may impact the rate of change over time [27]. LGM was conducted on patients who underwent a surgical procedure across four time points. Furthermore, to assess heterogeneity within the sample multigroup, LGM models were conducted across sex (i.e., males, females) and age groups (i.e., adolescents < 18 years of age; emerging adulthood = 18–25 years of age; young adulthood = 26–40 years of age; middle adulthood = 41–65 years of age; older adulthood = 66 years of age and older).

## 3. Results

Of the 3920 cases, 2339 (59.67%) were missing more than 10% of the IKDC-6 at one of four time points and were deleted. Only one case (i.e., 0.06%) was missing only one item from the IKDC-6 across time points and that case was retained, with the missing value replaced with mean imputation [9]. A total of 77 univariate and multivariate outliers were identified and removed from the dataset, leaving a total of 1504 cases for analysis across the four time points. The sample consisted of 688 males (45.7%) and 672 females (44.7%) with a mean age of 32.19 ± 14.60 years (Table 1). The mean score for the IKDC-6 across time points and by sex and age is reported in Table 2.

### 3.1. Latent Growth Modeling

The 1504 participants who completed the IKDC-6 at all four time points were used to conduct an LGM. Rate of growth was assessed by testing both linear and quadratic growth, and linear rate of change demonstrated a superior fit (CFI = 0.997; χ^2^ (5) = 49.126, *p* < 0.001; TLI = 0.968; IFI = 0.973; SRMR = 0.026, RMSEA = 0.077); thus, assessment of the intercept and slope parameters for the linear models was permitted [9,27]. The intercept values (M = 46.24, *p* < 0.001; variance = 68.97, *p* < 0.001; Table 3) indicated that, on average, participants reported moderate disablement at the initial baseline visit and a statistically significant intercept variance indicated individual differences at baseline. The positive, statistically significant slope mean value (M = 9.81, *p* < 0.001) indicated that, on average, knee health increased over time, and a significant variance (variance = 10.80, *p* < 0.001) indicated that individual knee health scores changed at different rates over time. Lastly, there was a statistically non-significant covariance (4.44, *p* = 0.148) which indicated that initial scores on knee health did not influence the rate of change in knee health scores over time.

### 3.2. Multigroup Latent Growth Modeling

Individuals who reported sex and age were used for the multigroup LGM. Individuals in the older adulthood group (66 years of age and older) were not included in the multigroup LGM for age groups because of the limited sample size (n = 32). Multigroup LGM results indicated that participants responded similarly to the IKDC-6 preoperatively, reporting poor knee health. Both male and female and all age groups perceived knee health improved over time (i.e., increased scores on the IKDC-6). Males showed a slightly faster rate of change than females (Table 3), but the covariance was not statistically significant, indicating that preoperative scores on the IKDC-6 did not significantly influence the rate of change over time between males and females. Adolescents (<18 years of age) demonstrated the fastest rate of change (M = 13.98), and the negative covariance indicated that those who scored lower on the IKDC-6 preoperatively increased at a faster rate; however, the covariance was not statistically significant, indicating that initial scores on the IKDC-6 did not largely influence the rate of change overtime. Emerging adults (18–25 years) had the second fastest rate of change and had a small, non-significant covariance (−0.64, *p* = 0.92), indicating that preoperative scores on the IKDC-6 did not significantly influence the rate of change over time (Table 3). Young adults (26–40 years) and middle-aged adults (41–65 years) changed at similar rates and both had positive covariances which indicated that those who scored higher on the IKDC-6 preoperatively increased at faster rates; however, the *p*-values for these age group were statistically non-significant.

## 4. Discussion

The purpose of this study was to assess LGM characteristics (i.e., rate of change) of the IKDC-6 in a sample of patients who underwent knee arthroscopy and completed the IKDC-6 at multiple visits (i.e., preoperatively, 3 months, 6 months, and 12 months postoperatively). We investigated if preoperative patient-reported scores influenced the rate of change in perceived knee health over time following surgical intervention. Additionally, the heterogeneity in individual responses on the IKDC-6 across subgroups (i.e., age and sex) was assessed using LGM analysis. Our results indicate that initial scores did not influence the rate of change in knee health scores over time; however, on average, perceived knee health increased over time, and individual knee health scores changed at different rates over time. We also found that the adolescent age group (<18 years) had the fastest recovery rate.

We hypothesized that the IKDC-6 could be used as a prognostic tool if initial scores on the IKDC-6 influenced the rate of change in perceived knee health. For example, if patients with initially worse knee health scores had slower rates of recovery after surgery, clinicians may consider this information, along with pathology and surgery type, to guide intervention selection and rehabilitation design or to inform patient communication. Our findings, however, demonstrate that preoperative scores did not influence the rate of change in perceived knee health over time, suggesting that the selected surgical approach generally produced favorable outcomes and that improvements in perceived knee health were generally experienced irrespective of preoperative knee impairment levels, knee pathology, or surgical intervention.

While the IKDC-6 may not be used as a prognostic tool as initially hypothesized, our results provide novel insights, showing that differences observed in age and sex may be a byproduct of another variable. Specifically, participants perceived their knee health to improve over time regardless of perceived knee health at baseline. Given that differences existed in the multigroup LGM analyses in a relatively large heterogenous sample, it is possible that the different rates of recovery observed may be due to other non-measured psychosocial variables (e.g., social support systems, coping strategies, patient resilience, kinesiophobia levels), as it has been reported with group differences in prior studies [30,31]. However, the lack of other important variables (e.g., specific knee pathology, surgical approach, condition prevalence, activity levels) in the dataset limits the analyses that can be performed to fully understand these differences. Additionally, the dataset encompassed various arthroscopic surgeries (e.g., meniscus repair, ACL reconstruction), but did not include other relevant groups (e.g., arthroplasties). Therefore, more research is needed to examine other variables that may influence group differences.

Our findings also demonstrate that a linear rate of change was the best fit for our sample of IKDC-6 responses; however, our analysis used responses to the IKDC-6 up to a maximum time point of 12 months after surgical intervention. While important time points exist within 0–12 months post-surgical intervention [32], useful data points also exist past 12 months of recovery [33]. Different rates of trajectory were identified in the IKDC-SKF up to 24 months postoperatively in prior research: early starters (i.e., most improvement observed from preop to one year follow-up), late starters (i.e., most improvement observed between 1 and 2 year follow-up), and late sinkers (i.e., slight improvement at 1 year follow-up and a decline in scores between 1 and 2 year follow-up) for patients who underwent ACLR surgery [33]. Thus, responses from 0–12 months may have limited the results of our LGM analysis (e.g., the full recovery period may not have been measured for some participants) and a longer evaluation period may provide more insights into recovery trajectories; however, it is also possible that the different trajectories are more likely to be found when evaluating specific populations (e.g., ACLR) as opposed to a large, heterogenous population of patients who underwent arthroscopic surgery.

Patients undergoing surgical intervention are expected to perceive improvement in knee health over time [34,35], and our LGM results confirm that, on average, patients’ perceived knee health improved following surgery at a linear rate of change. Furthermore, three different rates of trajectory have been identified for the IKDC-SKF [33] using Growth Mixture Model (GMM) methods. Our findings are congruent with the prior findings of heterogeneity between individual scores on the IKDC-SKF and IKDC-6, but the linear trajectory used in our analysis was not supported by prior research. The difference in trajectories could be due to the length of data collection (i.e., 12 months vs 24 months) or use of the longer form of the IKDC-SKF compared to the IKDC-6, as the two instruments have slight variations in the information being collected due to item reduction from the original scale.

Our LGM analysis demonstrated that our participants changed at different rates. To better understand where these interindividual differences exist, multigroup LGM analysis was conducted between sex and age subgroups. Males had a slightly higher mean slope, meaning that they had a faster rate of recovery compared to females, but this difference is not likely to be clinically meaningful. Differences in outcomes between sexes are debated for ACLR, with low-certainty evidence for inferior self-reported outcomes among females [36]. Conversely, sex was found to be a predictor for worse scores on the IKDC-SKF and KOOS following ACLR, with females reporting worse outcomes [37,38]. However, multigroup invariance found that males and females scored similarly on the IKDC-6 [22]. Therefore, more research is needed to understand the complexity of the recovery process between males and females and what may or may not influence sex differences.

Minimal differences have been found in younger patients compared to older patients who have undergone knee arthroscopy, with recovery profiles looking similar apart from pain recovery being significantly better in the older patients [39]. Additionally, previous researchers have found similar recovery patterns amongst different age groups (i.e., <20 years, 24–45 years, >50 years), with most patients having 90% of their muscle strength restored 3.4 weeks after arthroscopic surgery [40]. While these prior findings align with our findings of similar scores at baseline and all subgroups perceiving improvement across time, our analysis focused more on the rate of recovery. In this case, we did observe differences in age groups, with the two younger age groups perceiving recovery at faster rates than the two older age groups.

Social support may explain some of these differences, as it is one of the most adaptive factors for the recovery process [41] and younger aged individuals may have more social support from parents, coaches, teammates, and other groups compared to older individuals who may be less likely to be a part of support groups (e.g., team sport participation). Further, various knee abnormalities (e.g., osteophytes, cartilage damage, ligamentous damage, and osteoarthritis) tend to increase with age [42]. Studies indicate that at least 85% of individuals aged 50 years or older exhibit changes in articular cartilage in at least one knee compartment [43]. This prevalence is notably lower among younger age groups, with only 32% of those aged between 20 years and 29 years showing such changes and merely 13% of individuals aged 20 years or younger demonstrating similar abnormalities [43]. Thus, further research is needed to explain the nuances of differences in the recovery process across the life span.

### Limitations and Future Research

The SOS database serves as a valuable resource for clinical research due to its large sample size. However, it is crucial to recognize its limitations, particularly in terms of demographic variables. The lack of detailed demographic information (e.g., ethnicity, physical activity level, knee pathology, surgical procedure and approach, behavioral and mental health status, etc.) within the database hinders the ability to stratify groups based on these factors prior to LGM analysis. For example, without additional information, we are not able to understand how different knee pathologies and treatment interventions influence the rate of change over time, which is essential for optimizing clinical outcomes. Multigroup LGM analysis can provide valuable insights into these dynamics, allowing researchers to tailor interventions more effectively to individual patient needs. For instance, previous studies [33,44,45] have highlighted the significance of psychiatric history in predicting recovery following knee surgery. Higher levels of anxiety and depression have been associated with increased postoperative pain in Total Knee Replacement (TKR) procedures [46]. This association is so powerful that efforts are underway to develop biopsychosocial tools aimed at personalizing rehabilitation plans post-TKR [44].

Another notable limitation of the study is the relatively short duration of data collection, which only spanned 12 months. Studies have found that changes in IKDC scores can continue beyond 12 months, with some observations extending up to 28 months post-surgery [47]. Therefore, the findings of the analysis may not fully capture the long-term trends in patient outcomes, highlighting the need for longer-term follow-up assessments in future studies to provide a more comprehensive understanding of recovery trajectories. Therefore, additional research is needed with more comprehensive patient data (e.g., demographic variables, surgical procedure, etc.) over longer periods of time to better understand the recovery rate following arthroscopic surgery.

## 5. Conclusions

Our study assessed IKDC-6 characteristics in knee surgery patients, finding consistent postoperative improvements irrespective of preoperative scores. A linear rate of change was observed over 12 months, with heterogeneity in responses across demographics. Males exhibited a slightly faster recovery than females, and adolescents had the fastest recovery rate compared to other tested age groups. Future research should explore demographic and clinical factors impact on recovery, extending data collection beyond 12 months, for an enhanced understanding of the potential mechanisms that would explain the identified subgroup differences.

## Figures and Tables

**Table 1:** Demographics.

Characteristics	n	%
Sex		
Male	688	45.7
Female	672	44.7
Unknown	144	9.6
Age		
Adolescents	262	17.4
Emerging Adulthood	320	21.3
Young Adulthood	448	29.8
Middle Adulthood	368	24.5
Older Adulthood	32	2.1
Unknown	73	4.9

**Table 2:** Mean scores across time.

	Preoperative M (SD)	@3 Months M (SD)	@6 Months M (SD)	@12 Months M (SD)
Full Sample (n = 1504)	46.72 (17.76)	55.75 (12.67)	66.02 (14.40)	75.66 (17.53)
Adolescent (n = 262)	47.87 (19.00)	59.91 (12.26)	73.90 (12.78)	88.67 (13.52)
Emerging adulthood (n = 320)	49.32 (18.24)	56.69 (11.28)	67.93 (12.32)	80.2 (15.88)
Young adulthood (n = 448)	48.78 (17.42)	53.84 (11.87)	63.66 (13.40)	72.33 (16.23)
Middle adulthood (n = 368)	42.64 (16.38)	54.35 (13.25)	62.13 (14.67)	68.97 (16.20)
Male (n = 688)	48.23 (18.15)	56.55 (12.58)	67.59 (14.52)	77.86 (17.09)
Female (n = 672)	46.24 (17.41)	55.07 (12.52)	64.43 (13.64)	74.39 (17.61)

**Table 3:** IKDC Latent Growth Model parameters.

Subgroup	IMean	SMean	Covariance	*p*-Value
Full Sample	46.24	9.81	4.44	0.148
Adolescents (<18 years)	46.34	13.98	−8.15	0.285
Emerging Adult (18–25 years)	46.57	10.85	−0.64	0.921
Young Adult (26–40 years)	46.43	8.51	2.90	0.580
Middle Adult (41–65 years)	44.88	8.36	10.63	0.054
Male	46.90	10.27	3.94	0.390
Female	45.75	9.43	2.93	0.518

## Data Availability

The datasets analyzed during the study are not publicly available per study protocol; however, deidentified data may be available from the corresponding author with permission from the Cedar-Sinai Office of Research Compliance and Quality Improvement, the Kerlan-Jobe Institute, and the University of Idaho upon reasonable request.
